# Targeting the PAI-1 Mechanism with a Small Peptide Increases the Efficacy of Alteplase in a Rabbit Model of Chronic Empyema

**DOI:** 10.3390/pharmaceutics15051498

**Published:** 2023-05-14

**Authors:** Galina Florova, Christian J. De Vera, Rebekah L. Emerine, René A. Girard, Ali O. Azghani, Krishna Sarva, Jincy Jacob, Danna E. Morris, Mignote Chamiso, Steven Idell, Andrey A. Komissarov

**Affiliations:** 1 The Department of Cellular and Molecular Biology, University of Texas Health Science Center at Tyler (UTHSCT), Tyler, TX 75708, USA; galina.florova@uttyler.edu (G.F.); christianjordan.devera@uttyler.edu (C.J.D.V.); rebekah.emerine2@uttyler.edu (R.L.E.); rene.a.girard@uth.tmc.edu (R.A.G.); krishna.sarva@uttyler.edu (K.S.); jj12581@uttyler.edu (J.J.); danna.hill@gmail.com (D.E.M.); mc.etsub@gmail.com (M.C.); steven.idell@uthct.edu (S.I.); 2 The Department of Biology, University of Texas at Tyler, Tyler, TX 75799, USA; aazghani@uttyler.edu

**Keywords:** PAI-1, serpin, empyema, chronic, molecular target, preclinical, rabbit model, fibrinolytic therapy

## Abstract

The incidence of empyema is increasing and associated with a mortality rate of 20% in patients older than 65 years. Since 30% of patients with advanced empyema have contraindications to surgical treatment, novel, low-dose, pharmacological treatments are needed. A *Streptococcus pneumoniae*-induced rabbit model of chronic empyema recapitulates the progression, loculation, fibrotic repair, and pleural thickening of human disease. Treatment with single chain (sc) urokinase (scuPA) or tissue type (sctPA) plasminogen activators in doses 1.0–4.0 mg/kg were only partially effective in this model. Docking Site Peptide (DSP; 8.0 mg/kg), which decreased the dose of sctPA for successful fibrinolytic therapy in acute empyema model did not improve efficacy in combination with 2.0 mg/kg scuPA or sctPA. However, a two-fold increase in either sctPA or DSP (4.0 and 8.0 mg/kg or 2.0 and 16.0 mg/kg sctPA and DSP, respectively) resulted in 100% effective outcome. Thus, DSP-based Plasminogen Activator Inhibitor 1-Targeted Fibrinolytic Therapy (PAI-1-TFT) of chronic infectious pleural injury in rabbits increases the efficacy of alteplase rendering ineffective doses of sctPA effective. PAI-1-TFT represents a novel, well-tolerated treatment of empyema that is amenable to clinical introduction. The chronic empyema model recapitulates increased resistance of advanced human empyema to fibrinolytic therapy, thus allowing for studies of muti-injection treatments.

## 1. Introduction

For several decades, the incidence of empyema [1] steadily rose, together with mortality [2,3,4,5,6,7,8], in the United States, with an annual cost of care of up to five hundred million dollars [9]. According to the American Thoracic Society, empyema advances through three sequential stages-from early exudative (I) to fibrinopurulent (II) and finally to organization (III), which features adhesions sometimes leading to lung entrapment and a thick pleural rind. While the therapeutic goal, re-expansion of the affected lung, is achievable with thoracentesis in stage I, more advanced empyema (stage II) requires video-assisted thoracoscopic surgery (VATS) or intrapleural fibrinolytic therapy (IPFT) and stage III often requires thoracotomy [8,10,11,12,13,14,15]. Patients that present with extensive loculation, lung abscess, and pleural thickening are more likely to suffer failed IPFT [16,17,18,19,20,21] and require surgical intervention [19,22]. While thoracoscopic surgery is a widely accepted primary therapy for empyema [15], up to 30% of patients are poor candidates for either surgery or, alternatively, for conventional IPFT. Moreover, the cohort of patients who are treated only with simple drainage were a decade older and had higher mortality than those who are good candidates for the thoracoscopic surgery [14,23,24]. Pharmacological treatment of chronic empyema involves relatively slow fibrinolysis, and often fails [19,25,26]. Thus, preclinical research focused on the development and testing of novel, well-tolerated, pharmacological treatments that may complement surgery with effective alternatives to simple drainage for patients with chronic empyema. Recent advances in technology expanded the purview of less invasive VATS and IPFT to treat stage II and even stage III empyema [27,28,29,30,31], offering the predicate to explore novel, targeted, pharmacological approaches to treat more advanced/chronic empyema. Increasing the intrapleural half-life of fibrinolysins and the rate of fibrinolysis may address both intrapleural fibrin deposition and pleural thickening, thereby widening the therapeutic window for IPFT in empyema.

**Figure 1:**
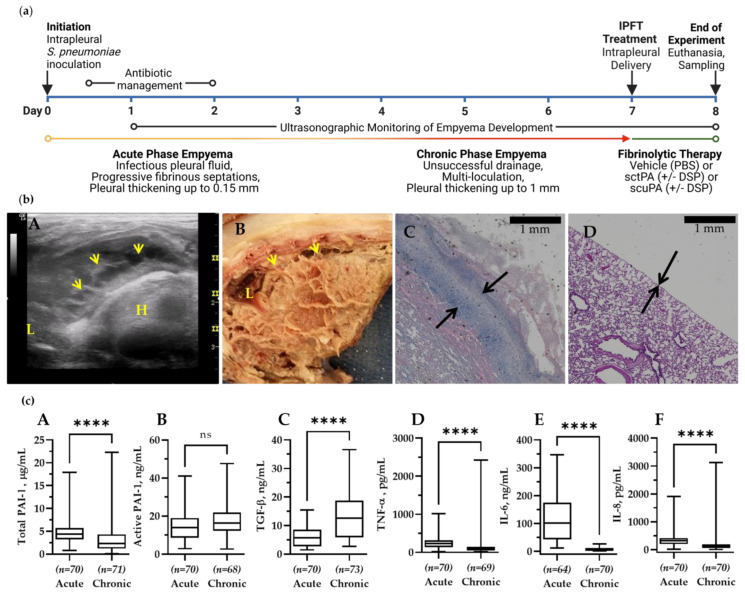
The Model of Chronic Empyema in Rabbits Features Severe Pleural Fibrosis Combined with Increased Pleural Thickening. (**a**) Schematic time course of initiation and development of chronic *Streptococcus pneumoniae* -induced empyema modeled in rabbits (0–7 d), followed by treatment (24 h)**.** Infectious pleural injury was initiated by intrapleural injection of *S. pneumoniae* at time zero. Progression of the pleural fibrosis was monitored by daily ultrasonography [41,42]. The acute phase (yellow) approaches its peak by 3 d. The chronic phase, which involves increased intrapleural organization (red), follows the acute phase. Both standard Intrapleural Fibrinolytic Therapy (IPFT) with sctPA or scuPA or PAI-1-TFT (green) were given at 7 d after initiation of empyema. Excess pleural fluid (up to 10 mL) was drained as needed at 8 h after treatment. Outcomes were assessed at 8 d (24 h after treatment) using ultrasonography and postmortem visualization (documented by photography). Samples of lung tissue were collected and stained by hematoxylin and eosin (H&E). A Gross Lung Injury Score (GLIS) [40,41,42], which ranges from 0 to 50 units, where a clear pleural space is 0; too numerous to count (TNTC) fibrin formations corresponds with 50, and GLIS ≤ 10 indicates successful treatment, was determined. GLIS is the sum of the number of intrapleural fibrin strands (1 unit each) and large nets and aggregates (5 units each). (**b**) (**A**) Chest ultrasonography of a *S. pneumoniae*-induced chronic empyema model at 7 d. The yellow arrows indicate intrapleural fibrin deposition; L = lung, H = heart; (**B**) postmortem visual evaluation with extensive adhesions, fibrinous coating of the lung, and multiloculated right hemithorax; (**C**) histologic assessment shows a pleural surface coated with fibrin, inflammation, and subpleural pneumonitis with increased organization. Pleural thickening (black arrows) surface to basement membrane (1 mm scale); (**D**) histologic assessment of the edge of a normal, uninjured lung with pleural thickening (black arrows) and a scale bar 1 mm. The visceral pleural surface is oriented at the bottom left portion of each panel. (**c**) Changes in the pleural fluid levels of PAI-1 and biomarkers of inflammation during the transition from acute to chronic empyema in rabbits. Samples of pleural fluid were withdrawn at 3 d (baseline acute, early stage) and 7 d (baseline chronic, advanced-stage) and levels of total (**A**) and active (**B**) PAI-1, TGF-β (**C**), TNF-α (**D**), IL-6 (**E**), IL-8 (**F**), and IL-1β were determined as previously described [41]. Statistical significance between these two sample groups was determined using an unpaired, 2-tailed Kolmogorov–Smirnov test. Statistical significance: **** and ns (not significant) denote *p* < 0.0001 and > 0.05, respectively. ELISA (R&D Systems, IN; Molecular Innovations, MI; and Ray Biotech, GA) were used to determine levels of proteins in pleural fluids.

Plasminogen activator inhibitor 1 (PAI-1) is overexpressed in pleural fluids in empyema by up to three orders of magnitude [32,33,34], and is the most effective mechanism-based inhibitor (serpin) of tissue (tPA) and urokinase (uPA) plasminogen activators [35,36]—therapeutics used for IPFT [37,38]. We hypothesized that neutralizing intrapleural PAI-1 pharmacologically would increase the efficacy of fibrinolytic therapy, and, thus, in a significant decrease in the dose of plasminogen activators needed for successful treatment. PAI-1-targeted fibrinolytic therapy (PAI-1TFT) is designed to decrease the effective dose of a fibrinolysin by protecting intrapleural plasminogen activating activity from PAI-1, thereby rendering otherwise ineffective doses of plasminogen activator effective. Our results demonstrate that targeting the PAI-1 mechanism increases the efficacy of single chain (sc) tPA (Alteplase) and scuPA in rabbit models of chemically induced pleural injury and acute, early-stage empyema to 8-fold [39,40,41]. *S. pneumoniae* induced empyema in rabbits [41,42] recapitulates the key features of empyema observed in humans, including the progression from an acute, early stage to a more advanced chronic one, accompanied by increasing pleural fibrosis, loculation, and pleural thickening (Figure 1, panel (a)). This empyema model features multi-loculation, significant pleural thickening, and high levels of PAI-1, the molecular target for our adjuncts. Administering 2 mg/kg sctPA or scuPA effectively resolved fibrinous pleural adhesions in acute, early-stage empyema in rabbits [41,42]. When a short docking site peptide (EEIIMD; DSP) was used in acute stage empyema, an up to 8-fold decrease in the minimal effective dose (MED; the minimal dose of a plasminogen activator that results in effective fibrinolytic therapy for every animal in a group (*n* = 5–6) [39,40,41,43]) was observed with sctPA, but not with scuPA [41].

In humans, delaying treatment leads to a late-stage, chronic empyema more resistant to IPFT, with increasing time to treatment correlating with increased failure of fibrinolytic therapy [18,20,21,44]. We evaluated fibrinolytic therapy with bolus injections of sctPA and scuPA alone and with DSP added during late-stage, chronic empyema in rabbits to (i) further validate a rabbit model of *S. pneumoniae* induced empyema [42], (ii) determine the MED of human sctPA and scuPA for this rabbit model of late-stage or chronic empyema, and (iii) demonstrate that DSP-based PAI-1-TFT increases the efficacy of plasminogen activators in a model of chronic, late-stage empyema in a manner similar to that observed in rabbit models of chemically induced pleural injury and acute empyema [41].

## 2. Materials and Methods

### 2.1. Animal Protocols

Animal procedures and techniques were approved by the Institutional Animal Care and Use Committee at The University of Texas Health Science Center at Tyler (IACUC protocols 616, 672)**.** New Zealand White rabbits (2.9–3.6 kg; average age 18 weeks) from Charles River Laboratories (Wilmington, MA, USA) were used for the model of chronic empyema. Female, pathogen-free animals (*n* = 46) were required for these experiments. An infectious pleural injury in rabbits was induced as described elsewhere [41,42]. Briefly, 1–5 × 10^8^ cfu of *S. pneumoniae* (D39 strain, National Collection of Type Cultures, Salisbury UK) in 3 mL of 0.5% brain-heart infusion agar (BD 238400, BD Diagnostic Systems, Hunt Valley, MD, USA) was injected into the right pleural space of an animal. Clavomox (10 mg/kg, subcutaneous, daily for 1–3 days as clinically indicated by Attending Veterinarian) (10000485, Zoetis, Parsippany, NJ, USA) was started at 28–30 h post-infection. Pleural injury and accumulation of loculation and pleural fluid was monitored by daily ultrasonography. PAI-1-TFT was administered via an 18-gauge catheter which was flushed with phosphate-buffered saline (0.5 mL). Samples of pleural fluids (≥0.5 mL) were collected prior to (baseline) and at 8 and 24 h after treatment [39,40,42,45]. Anesthesia, postoperative pain medication, and animal care were provided as reported previously [39,40,42,45]. Animals were monitored for signs of distress, pain, and worsening clinical status. In absence of these, rabbits were maintained for six days, treated on the seventh day, and euthanized on the eighth day so that the pleural space could be imaged, and fluid and tissue samples could be collected. Euthanasia was accomplished using intravenous injection of 1 mL of commercial euthanasia solution (sodium pentobarbital 390 mg/mL and phenytoin 50 mg/mL) followed by exsanguination. 

### 2.2. Ultrasonography

Development of chronic empyema was monitored via B-mode ultrasonography of the chest [39,46] using a Logiq e system (GE Healthcare, Milwaukee, WI, USA) equipped with version R5.2.x software and a multifrequency transducer model 12L-RS at a working frequency of 10 MHz, as previously reported [42].

### 2.3. Metrics of Treatment Efficacy and Pleural Injury

Gross Lung Injury Score (GLIS) was determined for each animal at 24 h after treatment (8th day). Post-euthanasia, the rabbit pleural cavity was opened, and pathological structures noted. GLIS varied from 0 (clear pleural space) to 50 (TNTC, too numerous to count), and equaled to a sum of fibrin strands (score 1 per each discrete strand), webs, sheets, and large (>5 mm) aggregates (score 5) as previously described [39,41,45]. Multiple visceral-parietal interconnected fibrin sheets and/or webs corresponded to GLIS = 50. PAI-1-TFT was considered successful if GLIS ≤ 10. Minimal effective dose (MED) is defined as the minimal dose of a plasminogen activator, alone or with PAI-1-targeting adjunct, which results in GLIS ≤ 10 for every animal in a group (*n* = 5–6). Morphometry was used to determine pleural thickening as reported elsewhere [42].

### 2.4. Docking Site Peptide

DSP (amino acid sequence: EEIIMD) was synthesized by GenScript (Piscataway, NJ, USA). 

### 2.5. ELISA for Quantitation of Antigens and PAI-1 Activity

ELISAs were used to determine levels of interleukin (IL)-6 (DY7984, R&D Systems, Minneapolis, MN, USA), tissue necrosis factor-α (TNF-α; (DY5670, R&D Systems, Minneapolis, MN, USA), transforming growth factor- β (TGF-β; (DY240-05, R&D Systems, Minneapolis, MN, USA), and IL-8 (ELL-IL8-1, RayBiotech, Peachtree Corners, GA, USA). Levels of active and total rabbit PAI-1 in pleural fluid were determined by ELISA (RbPAIKT; RBPAIKT-TOT, Molecular Innovations, Novi, MI, USA) according to the manufacturer’s protocol [41].

### 2.6. Plasminogen Activating and Fibrinolytic Activity Assays

Amidolytic plasminogen activation [39,47] and fibrinolytic [40,41,42,48] activities in baseline (time zero) pleural fluids were measured as previously described.

### 2.7. Histology

Tissue samples from rabbit lungs were paraffinized and sectioned to be stained. Hematoxylin and Eosin (H&E) (StatLab, Columbia, MD, USA) and Masson Trichrome (Thermo Fisher, Waltham, MA, USA) staining techniques were used to visualize pleural thickening and presence of collagen. Color bright field imaging at 4× were performed using Cytation 5 (Biotek, Winooski, VT, USA).

### 2.8. Immunofluorescence Staining and Imaging

Double-labeled immunofluorescence staining was performed on fixed and paraffin-embedded rabbit lung slides (5 µm) and on fixed rabbit empyema PF cultured on 8-well EZ slides (Millipore Sigma, Burlington, MA, USA) for 7 days. PAI-1, Fibrin(ogen), and nuclei were probed for using Alexa Fluor 488 (Jackson Immunoresearch, West Grove, PA, USA), Alexa Fluor 647 (Jackson Immunoresearch, West Grove, PA, USA), and Hoechst 33342 (Thermo Fisher, Waltham, MA, USA), respectively. Fluorescence imaging of the stained lung tissue slides were performed at 4× and 20× magnification using Cytation 5 (Biotek, Winooski, VT, USA). Z-stack imaging was performed on the stained 3D ex vivo culture slide at 40× magnification using Cytation 5 (Biotek, Winooski, VT, USA) as well.

### 2.9. Data Analysis and Statistics

Levels of statistical significance for non-pairing groups greater than two were determined using a two-tailed Kruskal–Wallis test with Dunn’s multiple comparison test. The level of statistical between two nonpaired groups were determined via two-tailed Kolmogorov–Smirnov for cumulative distribution and two-tailed Mann–Whitney test for cumulative ranks. A paired *t*-test was used to determine the statistical significance of paired data. Data analysis was performed using GraphPad Prism 9.3.1 as previously described [39,40,41,42].

## 3. Results

### 3.1. Chronic Infectious Pleural Injury in Rabbits Recapitulates Advanced-Stage Empyema in Humans

**Figure A1:**
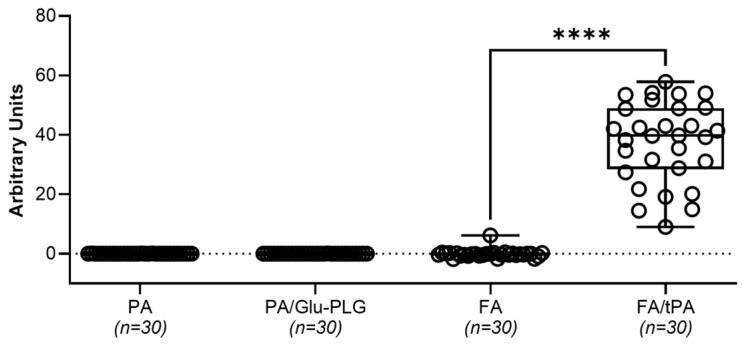
Plasminogen-activating (PA) and fibrinolytic (FA) activities in baseline pleural fluids of chronic empyema model. Plasminogen-activating (PA/Glu-PLG) and fibrinolytic (FA) activity was measured in samples of pleural fluid (5.0 µL; *n* = 30) collected at the baseline (time zero) prior to intrapleural injection of fibrinolytic therapy. Samples of 5 mL of pleural fluid were tested for Amidolytic plasmin activity with (PA/Glu-PLG) or without (PA) Glu-plasminogen, in clear 96-well flat bottom plates from Costar (Corning Inc., Corning, NY, USA) using a Synergy^TM^ HT Hybrid Reader (BioTek, Winooski, VT, USA). Plasmin activity was estimated from the initial slopes of the traces of the change in absorbance at 405 nm over time. A_405_ changed as a result of hydrolysis of chromogenic plasmin substrate (0.5 mM) added to the reaction mixture containing 5.0 µL of pleural fluid in 0.1 mL of 20 mM Hepes/NaOH buffer (pH 7.3, 0.16 M NaCl, 1.0 mg/mL BSA), as described previously [39,47]. The plasminogen activating (PA) activity was determined from rates of activation of exogenous Glu-plasminogen (200 nM) added to the reaction mixture containing 5.0 µL of pleural fluid in 0.1 mL of 20 mM Hepes/NaOH buffer (pH 7.3, 0.16 M NaCl, 1.0 mg/mL BSA) and 0.5 mM of the chromogenic plasmin substrate as described elsewhere (17, 18). PA was calculated from the slopes of linear dependences of A_405_ from square of tctPA time (t^2^) (A_405_ = A [PA] t^2^+ B; where A and B are constants), and measurements of standards with known concentrations as previously described [39,47]. Fibrinolytic activity (FA) was measured using 96-well plates with FITC-fibrin film as previously described [40,42,43]. Fibrinolytic activity in pleural fluids was calculated from the slopes of an increase in the fluorescence emission at 512 nm (excitation at 490 nm) over time as the FITC-fibrin degrades. Fibrinolytic potential was determined as the difference between fibrinolytic activity in samples of pleural fluid at baseline measured with (FA/tPA) and without (FA) supplementation with 10 nM sctPA [40]. Statistically significant differences between outcomes were determined using a two-tailed, paired *t*-test; **** denotes *p* < 0.0001.

A model of chronic empyema (Figure 1) includes the progression of infectious pleural injury beyond the 4 d acute [41] stage up to 21 days after induction of injury, and features an increase in the severity of pleural fibrosis and pleural thickening [42]. An 8 d model (7 d development followed by 24 h treatment) of chronic empyema (Figure 1, panel (a)) was chosen to evaluate the efficacy of bolus injections of either scuPA or sctPA and determining the MED for each fibrinolysin. This model of advanced-stage, chronic empyema features robust intrapleural fibrin deposition, detected by ultrasonography (Figure 1, panel (b), A), and documented by photography during necropsy (Figure 1, panel (b), B). Notably, the severity of the intrapleural fibrin and collagen deposition prevented pleural fluid drainage and progressed further than in models of chemically induced pleural injury and acute, early-stage empyema [41]. Pleural thickening in this rabbit model of advanced-stage empyema was also increased when compared to acute empyema in rabbits and chemically induced pleural injury [41] and to naïve rabbit lungs (Figure 1, panel (b), C and D). The timeline of advanced-stage infectious plural injury development and treatment are shown in Figure 1, panel (a). Changes in baseline levels of PAI-1 (total and active) and biomarkers of inflammation (TGF-β, TNF-α, IL-6, and IL-8) in pleural fluids of animals with advanced-stage (7 d) pleural injury, when compared to the acute stage (3 d) are shown as box plots in Figure 1 (panel (c), A–F, respectively). Notably, while levels of total PAI-1, TNF-α, IL-6, IL-8 (Figure 1, panel (c)), and IL-1 β (below level of detection), at baseline in chronic empyema were lower (*p* < 0.05) than those in the acute stage, the level of active PAI-1 did not change significantly, and the level of TGF-β was elevated (*p* < 0.05) when compared to baseline in acute empyema in rabbits [41] (Figure 1, panel (c)). During the transition between acute (3 d) and chronic (7 d) empyema in rabbits (Figure 1, panel (a)), the levels of inflammatory markers TNF-α, IL-6, IL-8, and IL-1 β in the pleural fluid at baseline decreased by 2.5, 57, 2.1, and more than 100-fold, respectively. In contrast, the level of TGF-β increased by 1.5-fold, likely promoting further development of intrapleural fibrosis, and increasing pleural thickening (Figure 1, panel (b), C). While the level of total PAI-1 decreased by 5.6-fold, the level of active PAI-1 present in the pleural space did not change (Table A1). Thus, levels of both active and total PAI-1, our molecular target, in chronic, advanced-stage empyema in rabbits remains up to two orders of magnitude higher than those in uninjured animals. Levels of TNF-α, IL-6, IL-8 in chronic empyema model were also markedly higher than those observed in uninjured animals [49,50]. Moreover, levels of PAI-1, TGF-β, TNF-α, IL-6, IL-8 observed in pleural fluids of human patients with empyema and with parapneumonic effusions were also higher than those in the transudative pleural fluids and were comparable to the levels observed in the rabbit empyema model (Table A1). At the time of treatment (7 d; Figure 1, panel (a)), both fibrinolytic and plasminogen-activating (PA) activities in pleural fluids collected from animals were suppressed by overexpressed PAI-1 (Figure A1). However, supplementation of the pleural fluid with exogenous tPA resulted in fibrinolytic activity due to activation of accumulated endogenous plasminogen (Figure A1). There was no statistical difference in red (RBC) or white (WBC) blood cell counts in the pleural fluids from animals with acute and chronic phases of *S. pneumoniae*-induced pleural injury. Nevertheless, there was a statistically significant decrease (*p* < 0.05) in the neutrophil count, with a decrease in neutrophil-lymphocyte ratio noted in pleural fluids of chronic phase of empyema in rabbits. These changes in the degree of pleural organization and inflammation may affect the processing of fibrinolysins during IPFT of advanced-stage empyema and result in an increase in the time needed for effective fibrinolysis.

### 3.2. The Rabbit Model of Chronic Empyema Recapitulates the Decrease in the Efficacy of Fibrinolytic Therapy Observed in Humans with Advanced-Stage Empyema

**Figure 2:**
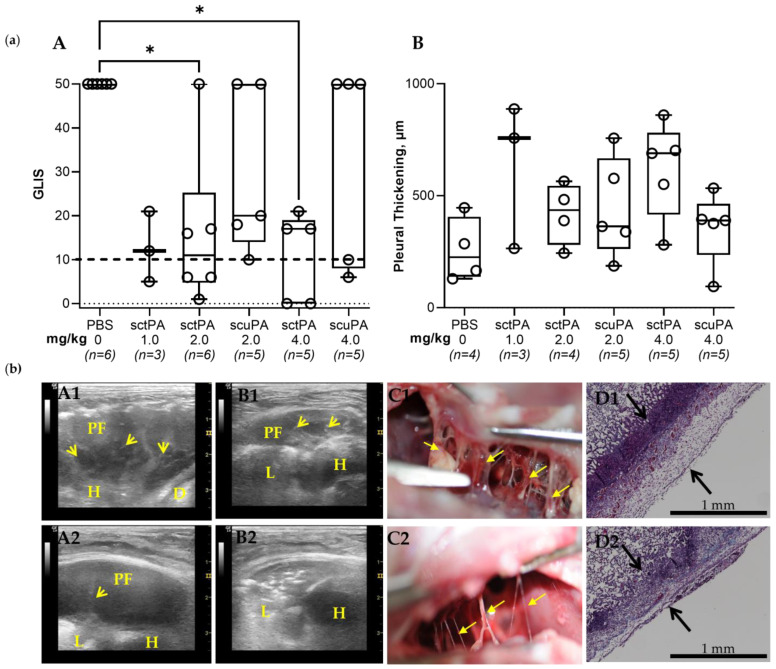
The Minimal Effective Dose (MED) for Treatment of Chronic Empyema in Rabbits with Bolus Injection of sctPA and scuPA is Higher than 4.0 mg/kg. (**a**) (**A**) Animals with advanced-stage infectious pleural injury were treated (from left to right) with: vehicle control (*n* = 6); 1.0 mg/kg sctPA (*n* = 3); 2.0 mg/kg sctPA (*n* = 6), 2.0 mg/kg scuPA (*n* = 5); 4.0 mg/kg sctPA (*n* = 5), 4.0 mg/kg scuPA (*n* = 5). The efficacy of fibrinolytic therapy was measured by GLIS (GLIS ≤ 10, at or below the dashed line, indicates successful outcome). The MED is the minimal dose of plasminogen activator which results in effective treatment for every animal in the group (*n* = 5–6). Statistically significant differences between outcomes were determined using Kruskal–Wallis test; * denotes *p* < 0.05. (**B**) Pleural thickening (µm) at 24 h after treatment (from left to right) with: vehicle control (*n* = 4); 1.0 mg/kg sctPA (*n* = 3); 2.0 mg/kg sctPA (*n* = 4), 2.0 mg/kg scuPA (*n* = 5); 4.0 mg/kg sctPA (*n* = 5), 4.0 mg/kg scuPA (*n* = 5). (**b**) Unsuccessful treatment with 4.0 mg/kg scuPA (GLIS = 50, **A1**–**D1**) and 4.0 mg/kg sctPA (GLIS = 17, **A2**–**D2**). Chest ultrasonography (yellow arrows—fibrin, L—lung, PF—pleural fluid; D—diaphragm, H—heart) at 24 h after treatment prior to (**A1,A2**) and after (**B1**,**B2**) drainage of the pleural fluid. A photograph of pleural fibrosis in situ during necropsy (**C1**,**C2**), and Trichrome staining (collagen is blue) of paraffin-embedded lung tissue at 4× magnification. Pleural thickening is shown with black arrows (surface to basement membrane) (**D1**,**D2**).

**Figure A2:**
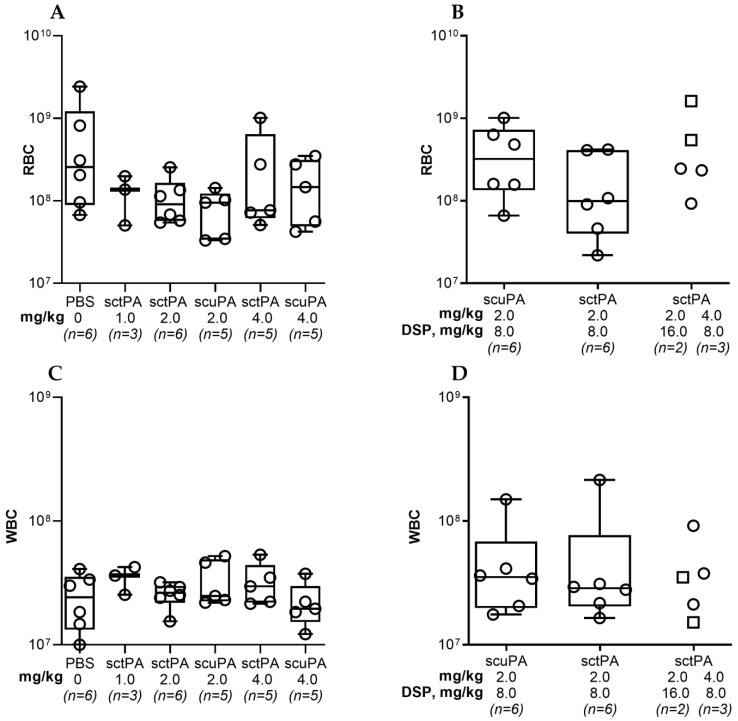
Red (RBC) and white (WBC) blood cell counts in pleural fluid of rabbits with chronic empyema at 24 h after IPFT with sctPA or scuPA (**A**,**C**) or with DSP-mediated PAI-1-TFT (**B**,**D**). RBC and WBC counts were determined as previously described [41]. (**A**) and (**C**)—animals were treated (from left to right) with: vehicle control (*n* = 6); 1.0 mg/kg sctPA (*n* = 3); 2.0 mg/kg sctPA (*n* = 6), 2.0 mg/kg scuPA (*n* = 5); 4.0 mg/kg sctPA (*n* = 5), 4.0 mg/kg scuPA (*n* = 5). (**B**,**D**)—animals were treated with (from left to right): sctPA 2.0 mg/kg with 8.0 mg/kg DSP (*n* = 6), scuPA 2.0 mg/kg with 8.0 mg/kg DSP (*n* = 6), sctPA 2.0 mg/kg with 16.0 mg/kg DSP (*n* = 2; squares), or sctPA 4.0 mg/kg with 8.0 mg/kg DSP (*n* = 3; circles). No statistically significant difference (*p* > 0.05) was detected between levels of RBC or WBC using two-tailed Kruskal–Wallis test with Dunn’s multiple comparison test.

**Figure A3:**
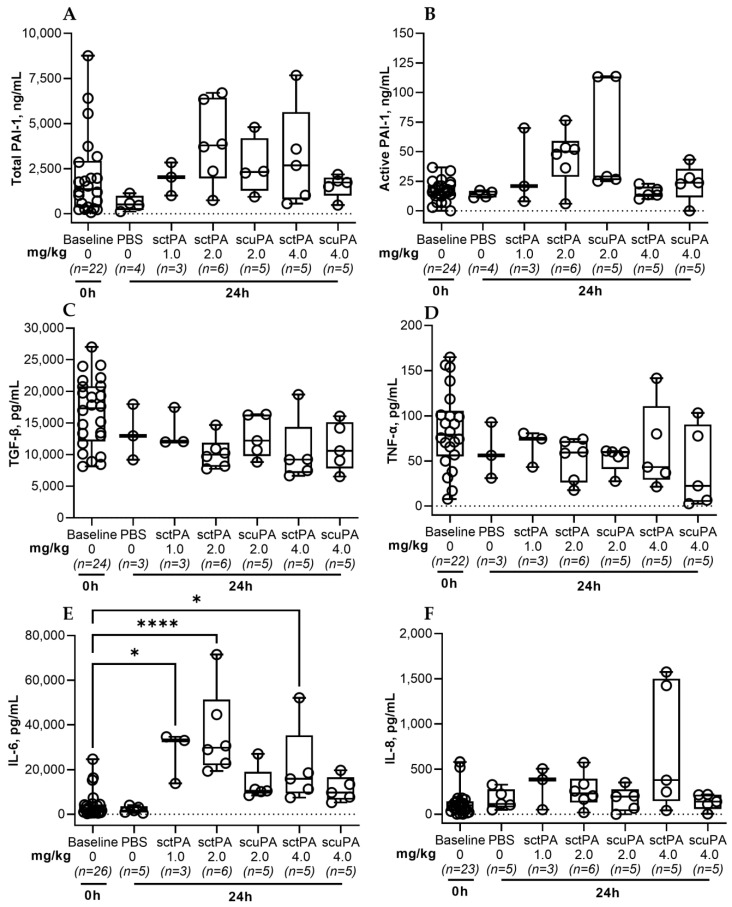
Levels of PAI-1 and Inflammatory biomarkers in pleural fluid of rabbits at baseline and 24 h after IPFT with sctPA or scuPA. Samples of pleural fluid were collected from animals with advanced-stage infectious pleural injury (from left to right) at the baseline (*n* = 22–26), and at 24 h after treatment with vehicle control (*n* = 6); 1.0 mg/kg sctPA (*n* = 3); 2.0 mg/kg sctPA (*n* = 6), 2.0 mg/kg scuPA (*n* = 5); 4.0 mg/kg sctPA (*n* = 5), 4.0 mg/kg scuPA (*n* = 5). Levels of total (**A**) and active (**B**) PAI-1, TGF-β (**C**), TNF-α (**D**), IL-6 (**E**) and IL-8 (**F**) were determined as previously described [41]. Statistically significant differences between levels of biomarkers were determined using a two-tailed Kruskal–Wallis test with Dunn’s multiple comparison test; * and **** denote *p* < 0.05 and 0.001, respectively. ELISA (R&D Systems, IN; Molecular Innovations, MI; and Ray Biotech, GA) were used to determine levels of proteins in pleural fluids.

Since the level of the molecular target, PAI-1, in chronic (7 d) empyema in rabbits was significantly lower than that in the acute stage (Figure 1, panel (c), A), a dose at half of the MED determined in the acute model [41,42] was chosen as the starting dose for treatment of chronic empyema in this rabbit model. *S. pneumoniae* was injected into the pleural space on 0 d (Figure 1, panel (a)) and the progression of the infectious pleural injury was monitored using daily ultrasonography (Figure 1, panel (a)). Intrapleural bolus injection of human sctPA or scuPA was administered on 7 d. Samples of pleural fluid were drawn prior to (baseline) and at 8 h and 24 h after treatment. The efficacy of treatment of chronic empyema was monitored by ultrasonography with postmortem visualization (documented by photography) and histological analysis of the collected lung tissue (Figure 2, panel (b)). Gross Lung Injury Scores (GLIS) [40,41,42] were used to determine the efficacy of the treatment (Figure 2, panel (a), A). Successful treatment outcome had GLIS ≤ 10 [40,41,42]. A starting dose of 1.0 mg/kg sctPA (*n* = 3) was not efficacious as expected for the MED and the dose was escalated by two-fold (bolus injection, 2.0 mg/kg) of sctPA (*n* = 6) or scuPA (*n* = 5)). However, neither plasminogen activator demonstrated the efficacy required for the MED (Figure 2, panel (a)). In order to identify the MED for a bolus injection, the dose was doubled again to 4.0 mg/kg, which is comparable to doses of Alteplase [37] and Urokinase [51] used in clinical practice. However, neither sctPA (*n* = 5), nor scuPA (*n* = 5) resulted in a treatment outcome of MED (GLIS ≤ 10 for every animal in a group, *n* = 5–6) (Figure 2, panel (a), A). Treatments were 40% effective even at this increased (4.0 mg/kg) dose of fibrinolysins. Thus, the intrapleural fibrin formed over 7 d as the empyema progressed to a chronic stage was either effectively lysed or not in 24 h post-treatment with the same high dose of fibrinolysins. Examples of unsuccessful treatment with 4.0 mg/kg scuPA (GLIS = 50), and 4.0 mg/kg sctPA (GLIS = 17) are shown in Figure 2, panel (b) (top and bottom rows of images, respectively). While neither treatment identified the MED, there was a trend towards effective IPFT for sctPA with increased dose, which resulted in a statistically significant difference (*p* < 0.05 when compared to the vehicle control; Figure 2, panel (a)). There was no statistically significant difference (*p* > 0.05) in RBC or WBC counts in the pleural fluids at 24 h after treatments (Figure A2). Levels of PAI-1 (total and active) and inflammatory biomarkers in pleural fluids collected at 24 h after treatment are shown in Figure A3. These higher doses of treatment with a bolus injection of sctPA or scuPA approached the highest doses (5.0–20.0 mg per injection) of Alteplase and Urokinase, which are currently used clinically [37,38,51,52,53]. However, treatment of chronic, advanced-stage empyema with 12.0–13.2 mg of either fibrinolysin did not reach the efficacy of MED. Thus, this model of advanced-stage empyema recapitulates failure of IPFT with an increase in severity of empyema observed in humans, and calls for testing of the multiple injections of plasminogen activators, as is carried out in clinical practice [37,38,51,52,53]. Notably, similar to the acute empyema model [42], successful treatment with sctPA or scuPA did not result in a decrease in the pleural thickening (Figure 2, panel (a) B; panel (b) D1 and D2).

### 3.3. DSP-Based PAI-1-Targeted Fibrinolytic Therapy Increases the Efficacy of sctPA in a Rabbit Model of Chronic Empyema

**Figure 3:**
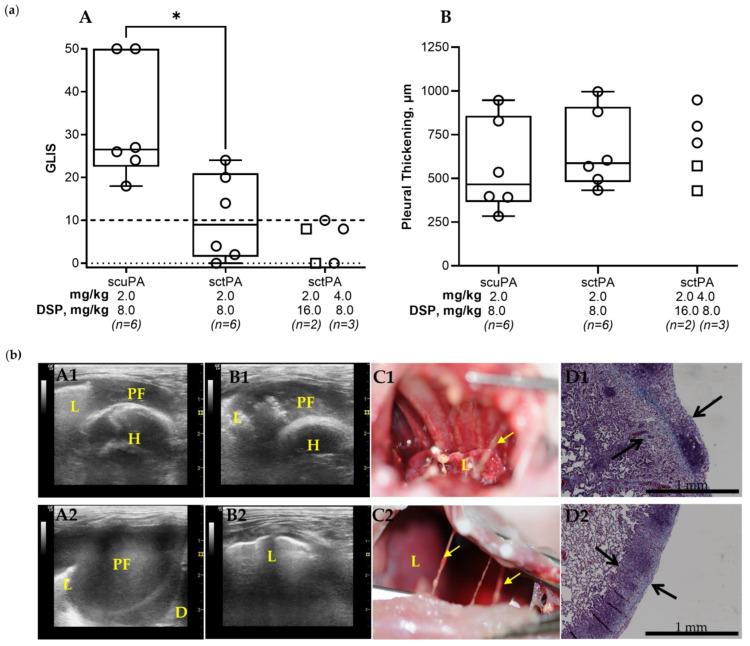
DSP Increases the Efficacy of Alteplase for Treatment of Chronic Empyema in Rabbits. (**a**) (**A**) Animals with empyema were treated with (from left to right): sctPA 2.0 mg/kg with 8.0 mg/kg DSP (*n* = 6), scuPA 2.0 mg/kg with 8.0 mg/kg DSP (*n* = 6), sctPA 2.0 mg/kg with 16.0 mg/kg DSP (*n* = 2; squares), or sctPA 4.0 mg/kg with 8.0 mg/kg DSP (*n* = 3; circles). The efficacy of fibrinolytic therapy was measured using GLIS (GLIS ≤ 10, at or below the dashed line indicates successful outcome). Statistically significant differences between outcomes were determined using Mann–Whitney test; * denotes *p* < 0.05. (**B**) Pleural thickening (µm) at 24 h after treatment (from left to right) with: sctPA 2.0 mg/kg with 8.0 mg/kg DSP (*n* = 6), scuPA 2.0 mg/kg with 8.0 mg/kg DSP (*n* = 6), sctPA 2.0 mg/kg with 16.0 mg/kg DSP (*n* = 2; squares), or sctPA 4.0 mg/kg with 8.0 mg/kg DSP (*n* = 3; circles). (**b**) Successful treatment with 4.0 mg/kg sctPA with 8.0 mg/kg DSP (GLIS = 8; **A1**–**D1**) and 2.0 mg/kg sctPA with 16.0 mg/kg DSP (GLIS = 8; **A2**–**D2**). Chest ultrasonography (yellow arrows—fibrin, L—lung, PF—pleural fluid; D—diaphragm, H—heart) at 24 h after the treatment prior to (**A1**,**A2**) and after (**B1**,**B2**) drainage of the pleural fluid; a photograph demonstrating the pleura at time of necropsy (**C1**,**C2**); and Trichrome staining (collagen is blue) of paraffin embedded lung tissue at 4× magnification. Pleural thickening is shown with black arrows (surface to basement membrane). (**D1**,**D2**).

Targeting the PAI-1 mechanism in chronic empyema allows for further validation of the approach and testing of the concept that PAI-1-TFT improves therapeutic outcomes in empyema. The efficacy of sctPA in an acute model empyema was increased by up to eight-fold using PAI-1-TFT with 8.0 mg/kg DSP [41]. Thus, 8.0 mg/kg DSP in combination with ineffective doses of 2.0 mg/kg sctPA or scuPA was tested to evaluate the effect of PAI-1-TFT on single bolus injection treatment of chronic empyema in rabbits (Figure 3, panel (a)). Administering 8.0 mg/kg of DSP in combination with 2.0 mg/kg sctPA showed a statistically significant improvement (*p* < 0.05) in outcome when compared to 8.0 mg/kg of DSP in combination with 2.0 mg/kg scuPA, demonstrating a trend to an increase in efficacy, when compared to 2.0 mg/kg sctPA alone (Figure 2, panel (a) A). Next, we hypothesized that a two-fold escalation of either the dose of DSP or sctPA would result in effective bolus PAI-1-TFT in chronic empyema. To test this hypothesis, animals were treated with either an ineffective dose of sctPA (2.0 mg/kg) in combination with a dose of DSP increased two-fold, to 16.0 mg/kg (*n* = 2) or with an ineffective dose of sctPA 4.0 mg/kg (two-fold increase) in combination with 8.0 mg/kg DSP (*n* = 3) (Figure 3, panel (a)). PAI-1-TFT with DSP (16.0 and 8.0 mg/kg) converted ineffective doses of sctPA (2.0 and 4.0 mg/kg, respectively) to effective ones (GLIS ≤ 10 for *n* = 5), supporting our hypothesis. However, similar to the acute empyema model [41] successful treatment with DSP in combination with sctPA (Figure 3, panel (a), A) was not accompanied with a decrease in the pleural thickening (Figure 3, (panel (a), B). Examples of successful treatment with 4.0 mg/kg sctPA combined with 8.0 mg/kg DSP (GLIS = 8) and 2.0 mg/kg sctPA with 16.0 mg/kg DSP (GLIS = 8) are shown in Figure 3, panel (b) (A1–D1 and A2–D2, respectively). Intrapleural fibrin accumulated over the course of 7 d development of chronic empyema (Figure 1, panel (b), B) was almost completely cleared at 24 h after treatment, indicating effective outcomes (Figure 3, panel (b), A-C). Effective treatment with PAI-1-TFT did not result in a decrease in the pleural thickening (Figure 3, panel (b), D). Thus, targeting the PAI-1 mechanism with DSP did not improve outcomes of scuPA treatment but rendered ineffective doses of sctPA effective. Previously, we demonstrated that PAI-1-TFT was effective for treatment of chemically induced pleural injury and in a model of acute (4 d), early-stage empyema [40,41,43] (Table 1). While DSP mediated PAI-1-TFT was effective with both sctPA and scuPA in treatment of chemically induced pleural injury, it was effective only with sctPA in acute and chronic stages of the infectious model (Table 1). However, MED for sctPA or scuPA alone in the chronic empyema model exceeds the doses currently used in clinical practice, thus bringing no advantage to further dose escalation experiments. There was no statistically significant difference (*p* > 0.05) in RBC or WBC counts in the pleural fluids at 24 h after treatment with DSP-based PAI-1-TFT (Figure A2).

**Figure A4:**
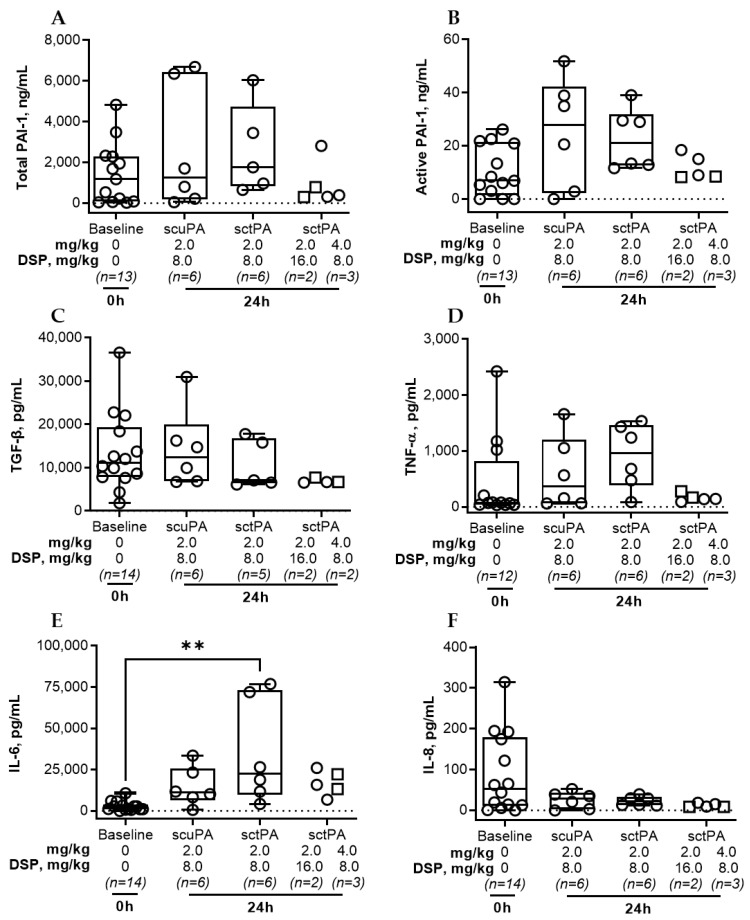
Levels of PAI-1 and Inflammatory biomarkers in pleural fluid of rabbits at baseline and 24 h after IPFT with sctPA or scuPA. Samples of pleural fluid were collected from animals with advanced-stage infectious pleural injury (from left to right): at the baseline (*n* = 12–14), and at 24 h after treatment with sctPA 2.0 mg/kg with 8.0 mg/kg DSP (*n* = 6), scuPA 2.0 mg/kg with 8.0 mg/kg DSP (*n* = 6), sctPA 2.0 mg/kg with 16.0 mg/kg DSP (*n* = 2; squares), or sctPA 4.0 mg/kg with 8.0 mg/kg DSP (*n* = 3; circles). Levels of total (**A**) and active (**B**) PAI-1, TGF-β (**C**), TNF-α (**D**), IL-6 (**E**) and IL-8 (**F**) were determined as previously described [41]. Statistically significant differences between levels of biomarkers were determined using a two-tailed Kruskal–Wallis test with Dunn’s multiple comparison test; ** denotes *p* < 0.01. ELISA (R&D Systems, IN; Molecular Innovations, MI; and Ray Biotech, GA) were used to determine levels of proteins in pleural fluids.

While levels of PAI-1 (total and active) and inflammatory biomarkers in pleural fluids collected at 24 h after treatment with DSP mediated PAI-1-TFT were not statistically significantly different (*p* > 0.05), there was a trend towards a decrease in each biomarker successful treatment with DSP combined with sctPA (Figure A4). Thus, targeting inflammation simultaneously with PAI-1 targeting may increase the efficacy of DSP mediated PAI-1-TFT in the chronic empyema model.

### 3.4. The Molecular Target, PAI-1, When Incorporated into a Fibrin Mesh, Contributes to the Failure of Fibrinolytic Therapy in Treatment of Chronic Empyema in Rabbits

**Figure 4:**
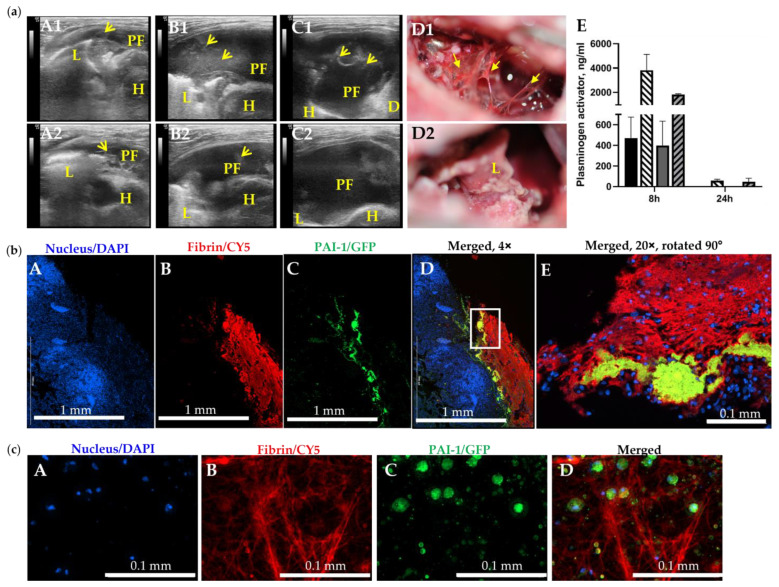
The Slow Rate of Intrapleural Fibrinolysis and PAI-1 Entrapped in Fibrin May Contribute to Failure of IPFT in Treatment of Chronic Empyema in Rabbits. (**a**) Failed (**A1**–**D1**) and successful (**A2**–**D2**) IPFT outcomes in rabbits with advanced-stage empyema were evaluated via ultrasonography (**A1**–**C1**,**A2**–**C2**) and gross imaging (**D1**,**D2**). Representative images of chest ultrasonography (**A1**,**A2**) immediately prior to (yellow arrows—fibrin, L—lung, PF—pleural fluid; D—diaphragm, H—heart ), (**B1**,**B2**) at 8 h after, and (**C1**,**C2**) at 24 h after treatment with bolus injection of sctPA (4.0 mg/kg), and gross images of the pleural space (**D1**,**D2**) with GLIS = 50 (**A1**–**D1**) and GLIS = 0 (**A2**–**D2**); (**E**) High levels of active plasminogen activator in samples of pleural fluid collected at 8 h after treatment with sctPA (black bar) or scuPA (gray bar) together with 6–8-fold higher levels of fibrinolysin antigens (hatched bars). At 24 h after treatment, plasminogen activating activity was completely suppressed (below level of detection), although antigen is still present. (**b**) PAI-1 is detectable in fibrin organizing in advanced-stage (7 d) empyema in rabbits. Fixed rabbit lungs with chronic empyema were paraffinized, sectioned, and attached to adhesive slides. Double-labeled immunofluorescence staining were performed, probing for nuclear stain (DAPI, Hoechst 33342), PAI-1 (GFP, AF 488), and Fibrinogen (CY5, AF 647). Imaging was performed at 4× (**A**–**D**) and 20× (**E**) magnification using Cytation 5. (**c**) Visualization of PAI-1 expressing activated mesothelial cells embedded in a fibrin scaffold. Chronic empyema pleural fluids collected from diseased rabbits were three dimensionally cultured in a sterile 8-well EZ slide (Millipore Sigma, Inc., Burlington, MA, USA) for up to 7 days. Double-labeled immunofluorescence staining was performed on the cells, as described. Z-stack imaging was performed at 40× (**A**–**D**) magnification using Cytation 5.

The *S. pneumoniae*-induced empyema model [42] recapitulates difficult pleural fluid drainage and thickened pleura associated with severe fibrosis in humans and requires comparable clinical management techniques [7,54,55,56,57,58,59,60]. The results of IPFT with single bolus injection of sctPA or scuPA (Figure 2; panel (a) A1 and A2, respectively) support the clinical notion that IPFT treatments for acute and advanced-stage empyema likely differ, with the latter having a higher chance of failure. Fibrin structure maturation and an increase in intrapleural scarring (substitution of fibrin with collagen) could adversely affect the rate of fibrinolysis and contribute to failure of IPFT. To test this hypothesis, we monitored intrapleural fibrosis and plasminogen activating activity in animals injected with the same bolus dose of sctPA (4.0 mg/kg) at 0, 8, and 24 h after treatment (Figure 4; panel (a), E). While at 0 and 8 h the presentation of the intrapleural fibrosis was similar for both animals (Figure 4, panel (a) A1,A2,B1,B2), the outcomes of the treatment were strikingly different (GLIS = 50, A1–D1 and GLIS = 0, A1–A2) (Figure 4, panel (a) C1,C2,D1,D2). There was no difference in the plasminogen activating activity in samples of pleural fluids withdrawn at 0 (Figure A1), 8, and 24 h (Figure 4, panel (a), E). Thus, the time of effective fibrinolysis (TEF, minimal time needed for endogenous fibrinolytic system to clear pleural space) in advanced empyema was indeed longer than the 4 to 8 h determined for chemically induced and acute empyema models [40,41,42,43,46]. Since both plasminogen-activating (Figure 4, panel (a), E) and fibrinolytic activities were suppressed at 24 h after treatment in pleural fluids of animals with both successful (GLIS ≤ 10) and unsuccessful (GLIS > 10) treatment, the TEF for chronic empyema was between 8 and 24 h. PAI-1 and fibrin were colocalized on the surface of the lung in chronic infectious pleural injury modes (Figure 4; panel (b)); this association could protect PAI-1 from immediate inactivation by an excess of plasminogen activator at IPFT. Thus, modifications of fibrin structure and a molecular target, PAI-1, entrapped in fibrin in chronic empyema may adversely affect the rate of intrapleural fibrinolysis resulting in failure of IPFT. Since further single bolus dose escalation (Figure 2, panel (a), A) in the model of advanced stage empyema could potentially increase bleeding, a strategy of multiple injection via a chest tube, similar to the approach used in clinical practice, may prove to be a more rewarding approach to be tested in future studies.

## 4. Discussion

PAI-1, which is elevated by up to three orders of magnitude in pleural fluid in empyema [32,33,34], was recently identified as a biomarker for septation severity and poor patient outcomes in empyema [44,61]. The goals of the present study were validation of a rabbit model of advanced-stage empyema and further testing of our central hypothesis, that PAI-1 is a useful molecular target in infectious pleural injury treatment. At time point 0, both fibrinolytic and plasminogen activating activity in pleural fluids from both humans [38,62] and rabbits [40,41,43,62] (Table A1) were suppressed by highly overexpressed PAI-1 [41,42], Figure 1c panels A, B as well as Figure A3 and Figure A4 (panels A and B). However, plasminogen accumulated in the pleural fluid, when activated to plasmin by tPA or uPA, produced fibrinolytic activity (Figure A1). *S. pneumoniae* pleural injury in rabbits [41,42] recapitulates key features of empyema in humans, including staging and timing, progressing from an early acute to more severe stage, which is accompanied by increasing pleural fibrosis, loculation, and pleural thickening. Validation of the *S. pneumoniae*-induced rabbit model of chronic infectious pleural injury is vital for further translation of preclinical observations to clinical trials in human patients. Demonstrating that the model mirrors the pathogenesis of empyema that was observed in clinical settings establishes the face validity of the model [63]. The model recapitulates advanced-stage empyema in humans, levels of intrapleural biomarkers of inflammation and PAI-1, showing increasingly severe pleural injury and pleural thickening in combination with resistance to bolus injection fibrinolytic therapy with high doses of sctPA and scuPA. Thus, the predictive validity of the model was established by treating animals with human plasminogen activators in doses comparable to those currently used in clinical settings [37,38,51,52,53]. Treatment with sctPA (1.0–4.0 mg/kg) was successful (GLIS ≤ 10) in approximately 50% of animals, and the efficacy of the highest dose of sctPA (4.0 mg/kg) was statistically different (*p* < 0.05) when compared to the vehicle control. In contrast to sctPA, scuPA (2.0–4.0 mg/kg) was markedly less effective. These results clearly support testing a multi-dose (multiple injections) approach to treatment with fibrinolysins to further increase the efficacy of IPFT and identify a MIMED (multiple injection minimal effective dose). Multiple intrapleural injections of Alteplase (sctPA) [37,52] and Urokinase [38,51,53] are currently used to treat empyema in humans. 

DSP is a short, negatively charged peptide (EEIIMD). Being part of the primed side of the reactive center loop of PAI-1, it presumably interferes with exosite interactions [64,65] between the 37-loop of the enzymes and PAI-1 [66,67]. Previously, we demonstrated that PAI-1-TFT with DSP or PAI-1 neutralizing mAbs increased the efficacy of sctPA up to eight-fold in two rabbit models of pleural injury (chemically induced and acute infectious) [40,41,43]. Here, we demonstrated that targeting the PAI-1 mechanism with DSP in a model of chronic, infectious empyema increases the efficacy of the treatment and brings the MED of sctPA with 16.0–8.0 mg/kg DSP to 2.0-4.0 mg/kg (Figure 3; panel (a) A) and predicts a better outcome if doses of both sctPA and DSP are elevated (to 4.0 and 16.0 mg/kg, respectively). In contrast to sctPA, DSP did not improve outcomes of fibrinolytic therapy with scuPA in either acute [41] or chronic (Figure 3, panel (a) A) empyema models. The higher affinity of tPA to PAI-1 compared to uPA, which depends on the protonation state of a histidine residue [68], may contribute to the striking difference between outcomes of DSP-mediated PAI-1-TFT with sctPA and scuPA in empyema models. The results of PAI-1-TFT with DSP and sctPA clearly demonstrate that the PAI-1-mechanism is a validated molecular target in both acute- [41], and chronic, advanced-stage (Figure 3) empyema modeled in rabbits. Previously, an increase in the MED of sctPA and an increase in the dose of DSP was required for 100% effective therapy (GLIS ≤ 10), which correlated with an increase in the level of PAI-1 in acute empyema, when compared to chemically induced pleural injury [41]. However, the decrease in the level of the molecular target in pleural fluid observed in chronic empyema, when compared to the acute stage (Figure 1, panel (c), A), did not result in a decrease in the MED of sctPA. The efficacy of both fibrinolysins (sctPA and scuPA) alone (Figure 2, panel (a), A) or in combination with DSP (Figure 3; panel (a), A) in the treatment of chronic empyema was markedly lower than in acute empyema [41]. Moreover, while the dose escalation of up to 4.0 mg/kg resulted in a statistically significant difference (*p* < 0.05) between sctPA-treated and vehicle control groups, the treatment outcome never approached the efficacy required by the definition of MED with any fibrinolysin. sctPA or scuPA alone or DSP-based PAI-1-TFT, successfully clears intrapleural fibrin deposition, it does not significantly impact the pleural thickening either in acute, early-stage [41,42] or chronic, advanced-stage empyema in rabbits. Likewise, treatments in the chronic empyema model caused no effect on pleural thickening when compared to vehicle controls at 24 h (Figure 2 panel (a), B; Figure 3 panel (a), B). Notably, this rabbit model of chronic empyema informs on possible limitations of PAI-1 as a biomarker of severity of intrapleural septation and poor outcomes [44]. While intrapleural levels of PAI-1 in a model of acute, early-stage empyema were higher than those in chronic, advanced-stage empyema (Figure 1, panel (c), A), the efficacy of both IPFT and PAI-1-TFT was markedly lower in treatment of the latter (Figure 2, panel (a); Figure 3; panel (a)) [41]. These results recapitulate advanced-stage empyema in humans [17,18,20], where IPFT fails more frequently due to slower fibrinolysis [20,21,30,69,70]. 

Thus, the rabbit model of *S. pneumoniae*-induced pleural injury recapitulates the evolution of untreated empyema from an acute to a more chronic stage with severe fibrosis, multiple loculation, pleural thickening, and a notable decrease in the efficacy of both IPFT and PAI-1-TFT. Compensating for slow fibrinolysis by increasing the dose of sctPA or scuPA (Figure 2, panel (a)), the frequency of multiple injections may result in only a slight increase in the efficacy, which may not compensate for the increased risk of bleeding complications. Indeed, treating patients with advanced-stage empyema using an “extended MIST2 protocol” (Alteplase 10 mg/DNase 5 mg, twice daily, for >6 doses) resulted in a four-fold increase in bleeding complications (10% compared to 2.5% using the standard protocol) [71]. Thus, the validity of our model is supported by its similarity to clinical presentation of advanced-stage empyema and responses to fibrinolytic therapy with human-tailored drugs, recapitulating findings observed in a clinical setting.

Two factors, the intrapleural half-life of active fibrinolysin and time needed for effective fibrinolysis, dramatically contribute to the outcome of pharmacological treatment of empyema [41,43,72]. Interestingly, the same combination of high intrapleural plasminogen-activating activity and progressing fibrinolysis, which is detected by ultrasonography at 8 h after treatment with a high dose of plasminogen activator, could be present at 24 h in both effective and ineffective outcomes (Figure 4, panel (a)). The transition of pleural injury from an acute to a chronic stage results in a decrease in PAI-1 and maturation of the intrapleural fibrin with the development of scarring and collagen deposition [73], together with incorporation of nucleic acids into fibrin structure [48], adversely affects the rate of intrapleural fibrinolysis. However, PAI-1 incorporated in fibrin may be protected from immediate inactivation by injected plasminogen activator (Figure 4, panel (b)), and it may play a critical role in the failure of IPFT in advanced-stage empyema, when more mature organization is observed. Thus, PAI-1-TFT affects the PAI-1 mechanism, protecting fibrinolysin from inhibition by any form of active PAI-1, increasing intrapleural half-life of the fibrinolysin, and supporting slow fibrinolysis. Therefore, PAI-1-TFT could be a reasonable alternative to dose escalation, compensating for the combination of slow fibrinolysis, rapid clearance of fibrinolysin from the pleural space, and constant replenishment of PAI-1 due to local overexpression. 

Table 1 demonstrates that (i) bolus injection MED for sctPA or scuPA alone in chronic, advanced-stage empyema in rabbits was higher than 4.0 mg/kg (average 13.2 mg which is comparable to doses using in clinical practice); (ii) DSP-mediated PAI-1-TFT increased the efficacy of sctPA and may convert ineffective doses of sctPA (2.0 and 4.0 mg/kg) to a MED (100% successful outcome in group of *n* = 5–6). Notably, the maximal decrease in MED by targeting the PAI-1 mechanism with DSP was eight-fold in both chemically induced and acute infectious models of pleural injury [41]. Moreover, the same eight-fold increase in the efficacy of scuPA was observed using mAbs-mediated PAI-1-TFT in chemically induced pleural injury [40] (Table 1). If eight-fold is the limit of efficacy increase and MED of sctPA for PAI-1-TFT with 16.0 mg/kg DSP is 2.0 mg/kg (Figure 3, panel (a) A; Table 1), the expected MED for sctPA alone in chronic empyema in rabbits could be as high as 16.0 mg/kg or more. This dose (~50 mg for bolus injection) is 5-fold higher than the dosage of Alteplase and Urokinase currently used in clinical settings and could induce significant intrapleural bleeding or even enter the bloodstream, activating the fibrinolytic system in the circulation. Novel, low-dose PAI-1-TFT may minimize the risk of bleeding complications and other adverse effects of high doses of plasminogen activator and allows treatment to be adapted to the population of patients with comorbidities and contraindications to conventional IPFT and surgery. These findings provide further support to our concept that novel strategies of targeting the PAI-1 mechanism could result in more effective clearance of pleural septations and improve therapeutic outcomes with lower doses of fibrinolysin [40,41,43].

## 5. Conclusions

Preclinical development of effective low-dose treatments for patients with chronic empyema, who decline or are not candidates for surgical intervention and are unable to receive conventional high-dose IPFT, could result in a paradigm shift in empyema management. The major conclusions from this work are:

1. Face and predictive validation of a novel model of chronic, advanced-stage empyema that closely recapitulates key physiological indicators seen in empyema in humans;
2. PAI-1 is a valid molecular target in *S. pneumoniae*-induced empyema modeled in rabbits, which closely recapitulates the key characteristics of this disease in humans;
3. Multiple injections of fibrinolysins could be tested in this model in order to develop and validate novel approaches to treatment of chronic empyema and expand treatment to patients who are not currently candidates for IPFT or surgery;
4. A new form of the molecular target, active PAI-1 that is incorporated into a fibrin mesh could contribute to slow fibrinolysis and failure of IPFT in treatment of advanced-stage empyema;
5. Preclinical development and testing of novel, low-dose PAI-1-TFT could result in innovative approaches to treating patients with advanced-stage, organizing pleural injury that fails drainage and is untreatable with surgery or high dose IPFT, as well as creating a foundation for translation to clinical trials.

Any improvement in non-surgical drainage by decreasing the dose of plasminogen activator, increasing the rate of intrapleural fibrinolysis, or increasing the half-life of plasminogen activating and fibrinolytic activities could positively affect the survival of patients with contraindications to or elevated risk from currently available interventions for chronic empyema. Even small improvements in the current treatment of advanced-stage empyema could result in a rise in efficacy and decrease mortality in this patient cohort.

## Figures and Tables

**Table 1:** PAI-1-Targeted Fibrinolytic Therapy (PAI-1-TFT) Decreases the Minimal Effective Doses (MED) when Compared with a Bolus Treatment with sctPA and scuPA Alone in Chemically Induced [40,41,43], Acute [41], and Chronic Infectious Pleural Injury in Rabbits.

Minimal Effective Doses *, mg/kg						
Model	Chemically Induced **		Infectious Acute ***		Infectious Chronic	
Treatment	Alone	PAI-1-TFT	Alone	PAI-1-TFT	Alone	PAI-1-TFT
sctPA	0.145	0.073	2.0	0.25	>4.0	≤2.0
scuPA	0.50	0.063	2.0	2.0	>4.0	>2.0

* A minimal effective dose (MED) is the dose of the plasminogen activator which results in 100% effective treatment (Gross Lung Injury Score; GLIS ≤ 10) for every animal in a group of 5–6; ** [40,41,43]; *** [41].

## Data Availability

All data are shared according to the NIH Resource Sharing statements per funded grants.

## Appendix A

**Table A1:** Levels of PAI-1, TGF-β, TNF-α, IL-6, and IL-8 in pleural fluid of rabbit empyema model and humans with complicated parapneumonic effusion/empyema, parapneumonic, and transudative pleural effusions.

	Pleural Disease	PAI-1 Antigen µg/mL	PAI-1 Activity ng/mL	TGF-β ng/mL	TNF-α pg/mL	IL-6 ng/mL	IL-8 ng/mL	Sample Size	References
Rabbit	Acute Empyema	5.18	16.93	12.68	174	65.54	0.25	*n* = 64–70	Figure 1c
	Chronic Empyema	0.92	15.53	18.76	70.87	1.15	0.12	*n* = 69–73	Figure 1c
Human	Complicated Parapneumonic Effusion/Empyema	0.70	40 **	x	x	x	x	*n* = 163	[32]
		x	x	x	x	x	4.74	*n* = 7	[74]
		x	x	x	1352	15	x	*n* = 11	[75]
		1.77	34.7 **	x	54	x	22.10	*n* = 25	[33]
		2 *	x	x	30 *	2.0 *	6.0 *	*n* = 17	[76]
		0.10	x	9.80	51.20	x	x	*n* = 19	[34]
		1.77	x	x	56.50	x	18.11	*n* = 30	[77]
		x	x	x	20.50	x	4.79	*n* = 12	[78]
		x	x	x	x	x	5.96	*n* = 51	[79]
		0.10	x	x	x	x	6.48	*n* = 22	[80]
		x	x	54.60	x	x	5.94	*n* = 11	[81]
		0.29	x	x	x	x	x	*n* = 27	[82]
		x	x	x	x	x	17.72	*n* = 20	[83]
		1.57	x	x	50.00	x	x	*n* = 53	[44]
	Parapneumonic Effusion	0.06	x	7.20	16.50	x	x	*n* =11	[34]
		x	x	x	x	15.30	1.88	*n* = 12	[78]
		x	x	x	90.20	22.80	x	*n* = 15	[84]
		0.15	x	x	34.90	x	x	*n* = 17	[85]
	Transudative Effusion	x	x	x	495	0.58	x	*n* = 17	[75]
		0.02	0.10	x	7	x	0.01	*n* = 25	[33]
		x	x	x	x	0.07	109	*n* = 9	[78]
		x	x	x	18.80	0.81	x	*n* = 15	[84]

** u/mL; * estimated from the data presented only as plots; x data not available.
